# Microbicidal Dispersions and Coatings from Hybrid Nanoparticles of Poly (Methyl Methacrylate), Poly (Diallyl Dimethyl Ammonium) Chloride, Lipids, and Surfactants

**DOI:** 10.3390/ijms20246150

**Published:** 2019-12-06

**Authors:** Rodrigo Tadeu Ribeiro, Carolina Nascimento Galvão, Yunys Pérez Betancourt, Beatriz Ideriha Mathiazzi, Ana Maria Carmona-Ribeiro

**Affiliations:** Biocolloids Laboratory, Departamento de Bioquímica, Instituto de Química, Universidade de São Paulo, Av. Prof. Lineu Prestes 748, 05508-000 São Paulo, Brazil; rodrigo@iq.usp.br (R.T.R.); y.betancourt@usp.br (Y.P.B.); bemathi@usp.br (B.I.M.)

**Keywords:** hybrid polymeric nanoparticles, biocompatible polymer, antimicrobial cationic polymer, films from casting of nanoparticles, dynamic light scattering, coatings wettability, bacteria and fungus viability, bactericidal activity of hybrid coatings, *Escherichia coli*, *Staphylococcus aureus*, *Candida albicans*

## Abstract

Hybrid and antimicrobial nanoparticles (NPs) of poly (methyl methacrylate) (PMMA) in the presence of poly (diallyl dimethyl ammonium) chloride (PDDA) were previously obtained by emulsion polymerization in absence of surfactant with low conversion. In the presence of amphiphiles such as cetyl trimethyl ammonium bromide (CTAB), dioctadecyl dimethyl ammonium bromide (DODAB) or soybean lecithin, we found that conversion increased substantially. In this work, the effect of the amphiphiles on the NPs core-shell structure and on the antimicrobial activity of the NPs was evaluated. NPs dispersions casted on silicon wafers, glass coverslips or polystyrene substrates were also used to obtain antimicrobial coatings. Methods for characterizing the dispersions and coatings were based on scanning electron microscopy, dynamic light scattering, determination of thickness, rugosity, and wettability for the coatings and determination of colony-forming unities (log CFU/mL) of microbia after 1 h interaction with the coatings or dispersions. The amphiphiles used during PMMA/PDDA/amphiphile NPs synthesis reduced the thickness of the NPs PDDA shell surrounding each particle. The antimicrobial activity of the dispersions and coatings were due to PDDA—the amphiphiles were either washed out by dialysis or remained in the PMMA polymeric core of the NPs. The most active NPs and coatings were those of PMMA/PDDA/CTAB—the corresponding coatings showed the highest rugosity and total surface area to interact with the microbes. The dispersions and coatings obtained by casting of the NPs dispersions onto silicon wafers were hydrophilic and exhibited microbicidal activity against *Escherichia coli*, *Staphylococcus aureus*, and *Candida albicans*. In addition, a major effect of reduction in particle size revealed the suitability of nanometric and cationic NPs (sizes below 100 nm) represented by PMMA/PDDA/CTAB NPs to yield maximal microbicidal activity from films and dispersions against all microbia tested. The reduction of cell viability by coatings and dispersions amounted to 6–8 logs from [PDDA] ≥ minimal microbicidal concentration.

## 1. Introduction

Several hybrid materials can exhibit antimicrobial activity [1,2,3]. For example, in restorative dentistry, inorganic antibacterial nanoparticles (NPs) incorporated to resin composites effectively reduce microorganism biofilm formation [4]. Examples of inorganic NPs with intrinsic antimicrobial activity are those made of ZnO, MgO, CuO, metallic silver, or gold, metal hydroxides such as Mg(OH)_2_ [5]. In addition, colloidal NPs of biodegradable chitosan, lignin or dextran [5,6] or biocompatible poly (methacrylate)- and poly (acrylate)-based copolymers can be loaded with antibacterial agents [7]. The other class of hybrid nanomaterials encompasses the biomimetic antimicrobial NPs and coatings obtained from the assembly of polymers, surfactants, or lipids [8,9] or from the use of virus-like, bacteria-like, or biological structure-like nanomaterials carrying antimicrobials [10].

The variety of biomedical applications for antimicrobial NPs range from coating implants or catheters to drug delivery [11,12,13]. The very useful NPs can yield a variety of nanostructures. For example, NPs may form hybrid antimicrobial coatings and films [8,14,15,16,17,18].

Antimicrobial polymeric NPs of poly (methyl methacrylate) (PMMA) synthesized in the presence of the cationic antimicrobial polymer poly (diallyl dimethyl ammonium) chloride (PDDA) were first obtained in 2015 joining the biocompatible character of PMMA with the microbicidal property of the cationic PDDA [19]. PMMA is non-biodegradable, non-absorbable, non-hemolytic and non-toxic with several biomedical applications in dentistry, ophtalmics, orthopaedics, pharmaceutics, etc. [20,21].

Early reports on methyl acrylate (MA) or methyl methacrylate (MMA) polymerization using more than 1.9 wt % of monomer mass in oil-in-water microemulsions resulted in phase separation during polymerization [22,23,24]. In emulsion polymerization, nucleation in the micelles or monomer droplets is followed by particles growth [25,26]. The initiator reacts with MMA in the micelles and with MMA inside the droplets in the aqueous phase, yielding oligo radicals that co-localize with the monomers and continue the polymerization. PDDA present during NPs synthesis in absence of surfactant possibly stabilized the smaller droplets of MMA yielding PMMA/PDDA hybrid NPs with sizes around 200 nm of mean hydrodynamic diameter [19]. Coatings prepared by spin-coating PMMA and dioctadecyl dimethyl ammonium bromide (DODAB) or PMMA and cetyl trimethyl ammonium bromide (CTAB) displayed good antimicrobial activity against bacteria upon contact. Whereas CTAB diffused from the coating to the outer medium to kill bacteria, DODAB killed bacteria upon contact because its affinity for PMMA was higher than the one of CTAB and DODAB remained in the PMMA matrix [27,28]. In dispersion, some antimicrobial activity was also reported for PMMA/DODAB or PMMA/CTAB NPs prepared by emulsion polymerization over a range of high concentration of the quaternary ammonium amphiphiles [29].

In our laboratory, the synthesis of hybrid PMMA/PDDA NPs in absence of amphiphiles, their core-shell structure and their antimicrobial activity were described and compared with the one of the free PDDA polymer [19]. However, the yield of polymerization was low and PDDA immobilization in the NPs reduced its activity [19]. Later on, the coatings obtained by casting PMMA/PDDA NPs onto different substrates and their activity against *Escherichia coli* and *Staphylococcus aureus* were reported [17]. In addition, the use of amphiphiles as stabilizers during the NPs synthesis was shown to improve substantially the yield for the polymerization for obtaining the PMMA/PDDA/amphiphile NPs [17]. In this work, the PMMA/PDDA, PMMA/PDDA/DODAB, PMMA/PDDA/CTAB and PMMA/PDDA/lecithin NPs are further studied to ascertain the amphiphile effect on the core-shell NP structure and its effects on antimicrobial activity of the dispersions and coatings.

The results show that PDDA as an outer shell of core-shell nanoparticles or simply as an additive to dispersions and films was the dominant element imparting the antimicrobial effect. The dispersions and coatings obtained by the casting of the NPs dispersions onto silicon wafers were hydrophilic and exhibited microbicidal activity against *Escherichia coli*, *Staphylococcus aureus* and *Candida albicans.* In addition, a major effect of reduction in particle size and increased rugosity for the coatings was associated with CTAB and its molecular geometry. CTAB promoted the synthesis of the smallest NPs. Their casting onto hydrophilic surfaces generated coatings with the highest rugosity and microbicidal activity. Nanometric and cationic NPs (sizes below 100 nm) of PMMA/PDDA/CTAB yielded maximal microbicidal activity for films and dispersions against all microbia tested amounting to 6–8 logs counting reduction from [PDDA] ≥ the minimal microbicidal concentration.

## 2. Results and Discussion

### 2.1. Physical and Antimicrobial Properties of PMMA/PDDA/Amphiphile Dispersions

**Figure 1:**
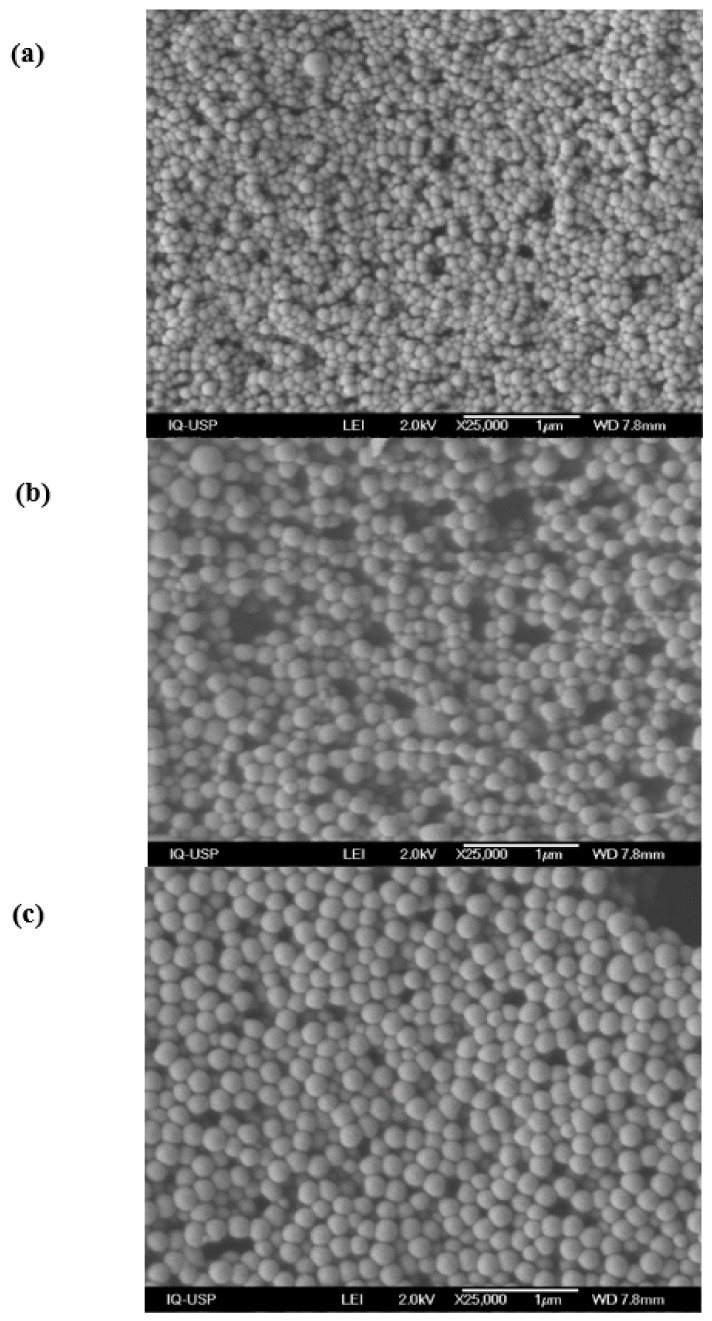
PMMA/PDDA/CTAB (**a**), PMMA/PDDA/DODAB (**b**) and PMMA/PDDA/lecithin coatings cast on silicon wafers from 50 µL droplets of nanoparticles (NPs) dispersions in water (**c**). The NPs were synthesized at 0.56 M MMA, 5 mg·mL^−1^ PDDA and 2 mM amphiphile (CTAB, DODAB, or lecithin). After synthesis, NPs were exhaustively dialyzed against water yielding final PDDA mass in the lyophilized coatings of 0.130 (**a**), 0.145 (**b**) and 0.140 mg (**c**). The bars represent 1000 nm.

SEM micrographs for the dried PMMA/PDDA/amphiphile dispersions after dialysis revealed uniform spherical NPs as synthesized in the presence of CTAB (Figure 1a), DODAB (Figure 1b) or lecithin (Figure 1c).

The micrographs on Figure 1 submitted to the ImageJ software yielded the mean diameter (D) of the dry NPs in the three dispersions (Table 1). As compared to the hydrodynamic diameter (Dz) obtained previously by dynamic light scattering (DLS) [17], the D values were smaller than the Dz ones for the NPs in water (Table 1).

The zeta-potentials (ζ) after dialysis remained positive, high and very similar to the ζ values for the PMMA/PDDA NPs obtained in absence of amphiphiles. The PDDA outer shell was responsible for the surface potential at the shear plane of the NPs in water dispersions (Table 1). In absence of PDDA, PMMA/DODAB, PMMA/CTAB, or PMMA/lecithin NPs exhibited zeta-potentials equal to 50, 26, and −21 mV, respectively [17]. In these cases, the amphiphile determined the surface potential at the shear plane of the NPs.

**Figure 2:**
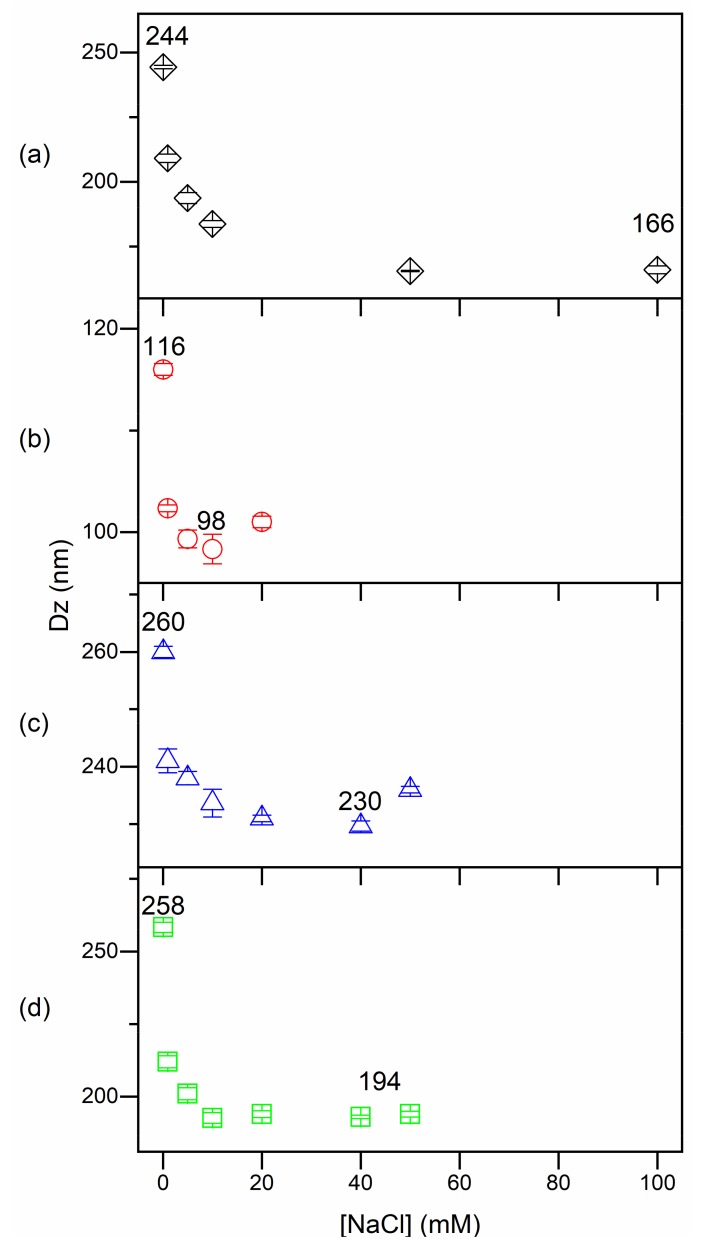
The collapse of the outer PDDA layer on PMMA/PDDA (**a**), PMMA/PDDA/DODAB (**b**), PMMA/PDDA/CTAB (**c**) and PMMA/PDDA/lecithin NPs (**d**) seen from Dz as a function of NaCl concentration.

Figure 2 shows the effect of NaCl on Dz of PMMA/PDDA/DODAB, PMMA/PDDA/CTAB, and PMMA/PDDA/lecithin NPs. The effect of increasing [NaCl] was decreasing Dz possibly due to decreasing the thickness of the outer PDDA shell in water and screening of the positive charges on the outer polymer chains (Figure 2). This was consistent with the drying effect on particle size shown in Table 1 with D being generally smaller than Dz. The collapse of the outer and stretched PDDA polymer shell can take place either by drying the NP (Table 1) or by increasing the ionic strength thereby causing the collapse of the outer shell (Figure 2). Consistently, minimum Dz values were equal to the mean diameter D of the dry particles obtained by SEM (Table 1; Figure 2). Above the minimum Dz, further increasing [NaCl] lead to loss of colloidal stability and NPs aggregation with a substantial increase in Dz due to NPs aggregation (not shown). This occurred above 50, 20 and 50 mM NaCl for PMMA/PDDA/DODAB, PMMA/PDDA/CTAB, and PMMA/PDDA/lecithin NPs (Figure 2). Therefore, these simple experiments and considerations allowed determining the thickness of the outer PDDA shell in the different NPs as (Dz–D)/2 or as (Dz–Dz_minimum_)/2. Furthermore, both determinations agreed (Table 1). Therefore, one can be confident that the shell thickness was properly obtained.

**Figure 3:**
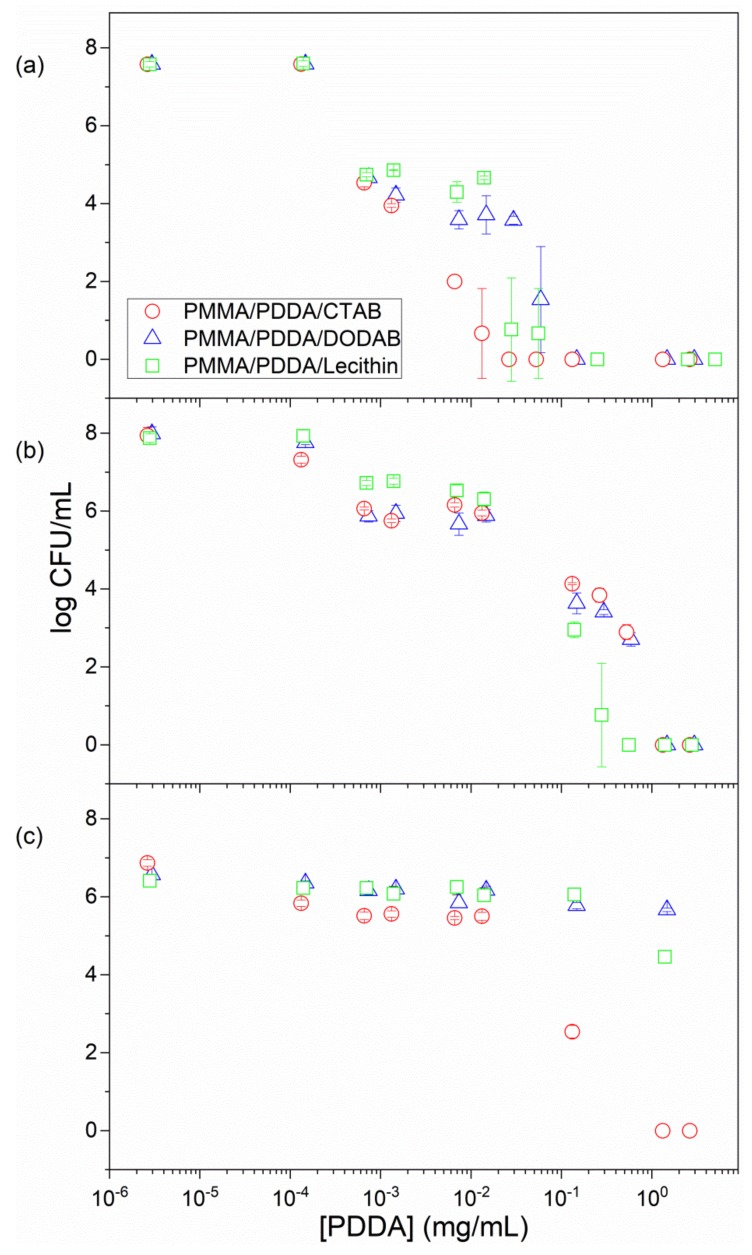
Antimicrobial activity of PMMA/PDDA/amphiphile dispersions after exhaustive dialysis against *E. coli* (**a**); *S. aureus* (**b**); *C. albicans* as a function of PDDA concentration (**c**). Cells and dispersions interacted for 1 h before plating for CFU counting.

Previous data for PMMA/PDDA NPs obtained in absence of emulsifiers as compared to the antimicrobial effect of PDDA alone revealed that both similarly killed *E. coli* and *S. aureus* but PDDA was much more effective against *C. albicans* than the PMMA/PDDA NPs [19]. For the coatings cast from PMMA/PDDA NPs onto glass coverslips at 0.25 mg PDDA, the tests against bacteria (*E. coli* or *S. aureus*) yielded complete loss of cell viability but tests against *C. albicans* were not performed [17]. In this work, the antimicrobial properties of PMMA/PDDA/amphiphile dispersions were obtained for bacteria and fungus over a range of PDDA concentrations (Figure 3). The minimal microbicidal concentration (MMC) determined for each dispersion was shown in Table 2. The real potency of the dispersions was established over orders of magnitude using a logarithmic scale for the cell viability as a function of PDDA concentration. Microbial viability decreased by 10^7^–10^8^ viable colony forming unities (CFU) upon interaction with the NPs for 1 h (Figure 3; Table 2).

From Figure 3 and Table 2, the most sensitive microorganism to the dispersions was *E. coli,* followed by *S. aureus* and *C. albicans.* The PMMA/PDDA/CTAB dispersions were the most effective dispersions killing the three strains by 7–8 logs in 1 h (Figure 3, Table 2). At this point, one should recall that all NPs exhibited hydrodynamic diameters of about 200 nm with the exception of the PMMA/PDDA/CTAB NPs with 90 ± 1 nm as Dz. Possibly, for this nanometric size especially; penetration of the NPs through the fungus cell wall and the cell membrane damaged these structures.

For other assemblies with PDDA, PDDA doses required for killing *S. aureus* were always higher than those required for killing *P. aeruginosa,* a gram-negative bacterium [14]. Tetraalkyl ammonium compounds have been recognized as efficient blockers of the potassium channels KcsA of the Gram-negative *E. coli* [30].

Whereas the Gram-negative bacteria are very sensitive to all cationic compounds and assemblies, the Gram-positive *S. aureus* is less sensitive via a mechanism of resistance. A sensor system for cationic antimicrobial molecules in *Staphylococcus* sp. causes resistance to cationic antimicrobial agents [31]. A short extracellular loop with a high density of negatively charged amino acid residues would attract and interact with cationic antimicrobial compounds. Transduction of this interaction signal would trigger the D-alanylation of teichoic acids and the lysylation of phosphatidylglycerol, resulting in a decreased negative charge of the cell surface and membrane, respectively. Thereby there would be a decreased attraction for cationic molecules.

**Figure 4:**
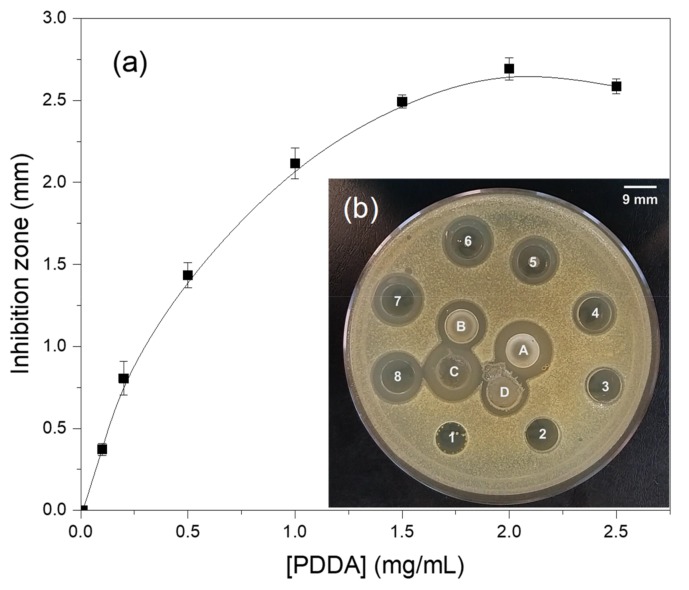
Inhibition zones against *S. aureus* as a function of PDDA concentration. From 1 to 8, 0.01, 0.2, 0.3, 0.5, 1,0, 1.5, 2.0 and 2.5 mg·mL^−1^ standard PDDA solutions were added to the 9 mm wells. In A, the PMMA/PDDA/DODAB dispersion before dialysis. In B, the same dispersion after dialysis (dilution factor of 2.8). In C, the supernatant of A. In D, the supernatant of B.

In order to evaluate the concentration of free and nanoparticle bound PDDA in the dispersions, the dispersions were dialyzed after synthesis and then centrifuged at 9,000 g in order to precipitate the NPs and separate them from their supernatant. The supernatant was then used to determine chloride concentration by micro-titration as previously described (see Methods). Since chloride is the PDDA counter-ion, its concentration is directly related to the PDDA concentration in mg·mL^−1^. Another possible way for evaluating [PDDA] in the supernatant was its biological activity determined from inhibition of growth on *S. aureus* seeded agar plates (Figure 4). The inhibition zones against *S. aureus* depended on PDDA concentration in the wells numbered from 1 to 8; increasing [PDDA], increased the inhibition zone though the curve was not linear. Nevertheless, it was possible to estimate [PDDA] over a range of low concentrations (0–1.0 mg·mL^−1^). After considering the dilution factor for the PMMA/PDDA/DODAB NPs after dialysis which was 2.8 the [PDDA] from the inhibition zone was 2.8 mg·mL^−1^ in reasonable agreement with the [PDDA] determined from chloride microtitration which was 2.5 mg·mL^−1^. One should notice that PDDA cannot permeate the dialysis membrane so that the PDDA in the dialyzed dispersions will still be available to contribute to the inhibition zones. DODAB also would not permeate the dialysis membrane if it were not bound to the PMMA matrix as described before [27]. Table 3 shows the [PDDA] in the supernatants of the 4 different NPs dispersions determined by the two methods: chloride microtitration and inhibition zones against *S. aureus.*

**Figure 5:**
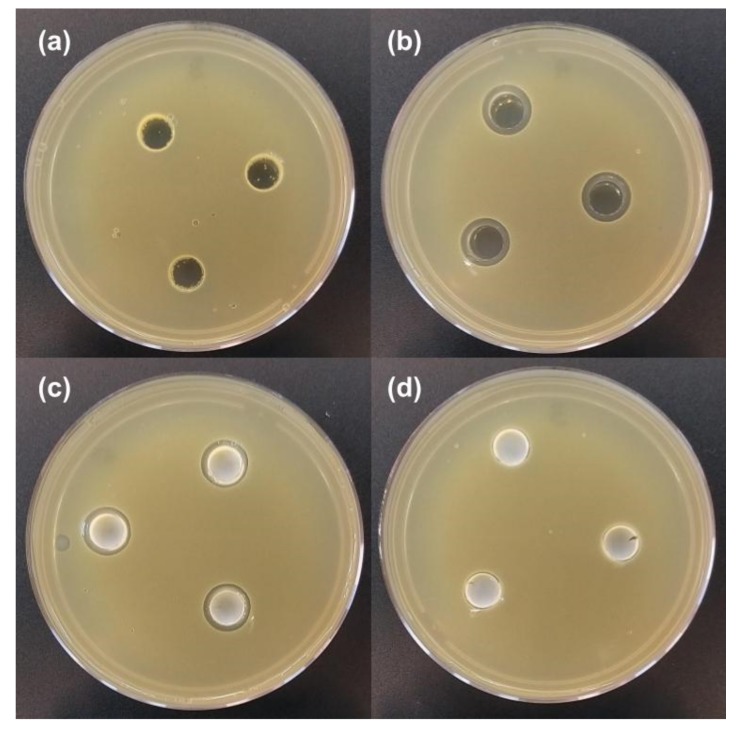
Inhibition zones against *S. aureus* for 0.1 mL of 2 mM DODAB (**a**), 2 mM CTAB (**b**); PMMA/CTAB NPs at 2 mM CTAB before dialysis (**c**) and PMMA/CTAB NPs after dialysis (**d**).

PDDA, DODAB and CTAB are all compounds with halides as counterions. Dispersions with DODAB or CTAB and PDDA may yield overestimated PDDA concentrations by the halide method. However, DODAB, despite not being permeable through the dialysis membrane, displayed high affinity for PMMA polymeric matrix and did not leach to the outer medium from PMMA coatings [27]. In addition, DODAB does not move through the agar and could not contribute to the inhibition zone observed for PMMA/PDDA/DODAB dialyzed NPs. Thus, for PMMA/PDDA/DODAB NPs after dialysis, both the microtitration and the determination of inhibition zones would give a reliable analysis of PDDA concentrations. On the other hand, CTAB was found to leach from PMMA coatings [27] and readily permeate the dialysis membrane (Figure 5). Thus CTAB would not be found in the supernatant of the dialyzed PMMA/PDDA/CTAB NPs—determination of PDDA concentration in the supernatant of dialyzed PMMA/PDDA/CTAB NPs would be reliable and not overestimated by the micro-titration method. Regarding the determination of inhibition zones, eventually, the lack of linear dependence of inhibition zones on [PDDA} would eventually yield poor determinations of concentration as indeed observed for PMMA/PDDA/lecithin dispersions. The composition of soybean lecithin includes negatively charged phospholipids and fatty acids [32], which would occupy the monomer droplet/water interface during PMMA synthesis eventually facilitating the incorporation of the positively charged PDDA in the PMMA NPs. However, the results on Table 3 show that the lecithin amphiphiles did not increase [PDDA]_nanoparticles_. In summary, the use of amphiphiles during PMMA/PDDA NPs synthesis did not improve PDDA attachment, entanglement, adsorption and/or mechanical immobilization to the NPs. It was in absence of surfactants, that the highest amount of PDDA became attached to the NPs yielding the most well-structured PDDA shell around the PMMA core (Table 1 and Table 3; Figure 2). In the presence of amphiphiles, by determining [PDDA] in the supernatant of centrifuged NPs it was found that most PDDA molecules became poorly attached to the NPs. The amphiphiles indeed reduced the interaction of PDDA with the NPs and diminished the thickness of the NP shell.

Figure 5 shows the different mobilities of DODAB or CTAB on *S. aureus* seeded agar plates. There was a lack of inhibition zones against *S. aureus* induced by 2mM DODAB in form of bilayer fragments [33] on Figure 5a. Inhibition zones occurred for 2 mM CTAB on Figure 5b and for PMMA/CTAB NPs at 2 mM CTAB before dialysis on Figure 5c. For PMMA/CTAB NPs after dialysis no inhibition zone was observed (Figure 5d). Whereas CTAB moved through the agar, DODAB bilayer fragments remained inside the limits of the sample wells. CTAB was no longer found in the PMMA/CTAB dialyzed NPs so that inhibition zones were not observed (Figure 5d).

**Figure 6:**
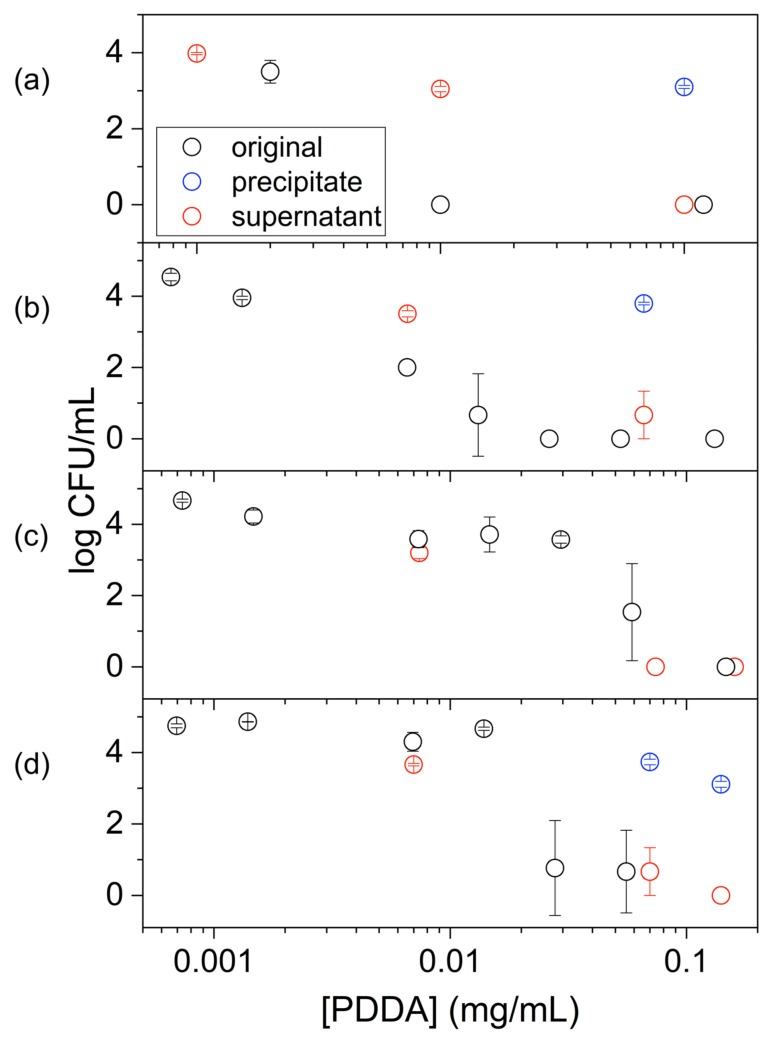
Activity of PDDA against *E. coli* in original dispersions (○), their precipitates (○) and supernatants (○). Dispersions are PMMA/ PDDA (**a**) or PMMA/PDDA/amphiphile where the amphiphile is CTAB (**b**), DODAB (**c**) or lecithin (**d**). Original dispersions are those obtained after exhaustive dialysis. Cells and dispersions or supernatants interacted for 1 h before plating for CFU counting. Dialysates were centrifuged (9000 g/40 min.) in order to separate NPs and supernatant for further separate the antimicrobial activity from the supernatant and the precipitate.

In order to confirm that the location of PDDA in the PMMA/PDDA dispersions was mainly in the water phase (Table 3), the antimicrobial activity against *E. coli* was determined in the supernatant of centrifuged dispersions and compared to the total activity of the original dispersions. Figure 6 shows this comparison for dispersions and their supernatants against *E. coli.* The activity of the supernatants was slightly smaller than or equal to the one obtained using the original dispersions. This result was consistent with the predominant location of PDDA in the water phase and its poor association with the PMMA/PDDA/amphiphile NPs. The best association between PDDA and PMMA was achieved in absence of surfactants.

### 2.2. Physical and Antimicrobial Properties of Coatings Prepared from PMMA/PDDA/Amphiphile Dispersions

**Figure 7:**
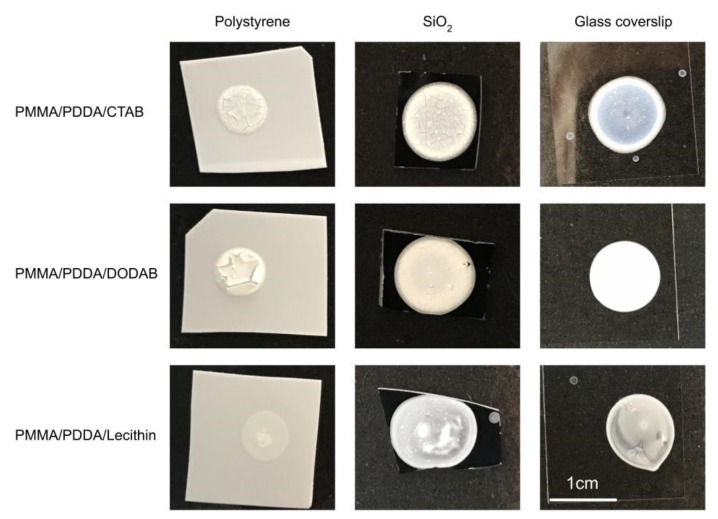
PMMA/PDDA/amphiphile films casted on three different surfaces (polystyrene, SiO_2_, or glass) from 50 µL droplets of NPs dispersions in water. The NPs were synthesized at 0.56 M MMA, 5 mg·mL^−1^ PDDA and 2 mM amphiphile, where the amphiphile was CTAB, DODAB, or lecithin. After synthesis, dispersions were exhaustively dialyzed. From top to bottom, PDDA amount was 0.130, 0.145, and 0.140 mg and PMMA amount was 1.3, 0.85, and 0.4 mg. The mass ratio PMMA:PDDA was 10, 6, and 3 from top to bottom. The bar corresponding to 1 cm on the bottom right holds for all films.

Figure 7 shows the macroscopic aspects of the PMMA/PDDA/amphiphile coatings obtained after casting and drying the NPs dispersions. The microscopic aspects of these coatings determined by SEM were also shown as the SEM micrographs previously shown as Figure 1. In general, their features were very similar to those previously described in detail for the PMMA/PDDA films in absence of amphiphiles on the three different substrates [17]. A uniform distribution of spherical nanoparticles of very similar size disposed in general side-by-side with some holes and discontinuities occurring at a low frequency showed the uniformity of the coatings.

The PMMA/PDDA/amphiphile coatings spread and adhered better to the hydrophilic surfaces such as the silicon wafers and the glass coverslips than to the hydrophobic polystyrene surfaces (Figure 7). The outer cationic PDDA shell on each NP interacted better with the anionic surfaces of silicon wafers or glass whereas the repulsion between the hydrophobic polystyrene surfaces; the hydrophilic PDDA shell of the NPs originated poor adherence and cracks on the PMMA/PDDA/amphiphile coatings (Figure 7). The coffee ring effect antagonized by the Marangoni effect was similar to effects previously described for coatings obtained from polymeric particles [34,35] as were the cracks visible for the coatings on the polystyrene surfaces [36]. Using lecithin as emulsifier resulted in some phase separation in the coatings that did not occur for CTAB and DODAB as emulsifiers (Figure 7).

In order to estimate the thickness of the coatings, simple calculations were performed as follows. The approximate area A for each coating on the hydrophilic substrates could be estimated considering spherical shape for the film with a radius of 0.5 cm: A = 0.785 cm^2^. The solid content for each dispersion given in Table 1 was previously determined gravimetrically [17] so that the total mass of solids for each coating obtained from 0.05 mL could easily be calculated for each film. Considering each coating as a cylinder its volume will be its base area multiplied by its height. From the radius for the dry particles and the PMMA density (1.18 g·cm^−3^), the volume and mass of each particle can be obtained. The total number of particles could be obtained from the total mass of the coating divided by the mass of one particle. The volume of the cylindrical films would then be given by the volume of each particle multiplied by the number of particles in each coating. Once known the volume of the cylinder, its thickness i.e., its height, could be estimated from the assumption of uniform coating and the division of the cylinder volume by its base area. From these simple calculations, the thicknesses for each film were estimated and added to Table 4.

The coatings of PMMA/PDDA/amphiphile obtained by casting of the NPs dispersions onto silicon wafers were also evaluated regarding their wettability from contact angle determinations (Table 4). These characteristics of the hybrid films compared to those of pure PMMA coatings revealed higher wettability for the hybrid coatings than the one determined for PMMA films (Table 4).

Coatings obtained by casting the PMMA/PDDA dispersions yielded lower contact angles than those obtained by spin-coating. Possibly, some molecules of the hydrophilic PDDA immobilized as an outer layer of the PMMA/PDDA nanoparticle imparted a more hydrophilic character to the film surface than the one of the spin-coated PMMA/PDDA (Table 4).

For coatings obtained by casting and drying the PMMA/PDDA/amphiphile dispersions onto the silicon wafers, there was a consistent decrease of hydrophobicity meaning a decrease of contact angles Ө_A_ in the following order: Ө_A_ DODAB > Ө_A_ lecithin > Ө_A_ CTAB (Table 4). The presence of residual amphiphile possibly at the surface of the PMMA core reduced the hydrophilicity of the coatings in the expected order from CTAB to lecithin to DODAB. The more hydrophobic amphiphile was DODAB which has a small hydrophilic polar head and double-chained saturated hydrocarbon tails. Lecithin has a mixed composition of phospholipids and fatty acids, some of them unsaturated [32] thereby displaying an intermediate hydrophobicity between the double-chained DODAB and the single-chained surfactant CTAB.

Rugosity (R) is a measure of small-scale variations of amplitude in the height of a surface given by the ratio between the real surface area (A) and the geometric surface area (A_g_) [37]. In the case of the NPs coatings from dispersions, R can be easily estimated due to the uniform size of the spherical NPs and the uniform general features of the coatings both microscopically (Figure 1) and macroscopically (Figure 7). The surface area for spherical NPs is given by 4Π(D/2)^2^, where D is the diameter of the dry NP. One needs to calculate the total number of NPs in a cross-section of the coating. The geometric area of the coating (A_g_) is 0.785 cm^2^. The film thickness divided by D yields the number of NPs layers per film. From the total number of NPs per coating divided by the number of NPs layers, the number of NPs per layer could be calculated. Since the area of the NPs is known multiplying this area per the number of NPs per layer divided by 2 will give the real surface area of the outer layer of the coating (A). Thus, R can be obtained from A/A_g_ (Table 4). The thicker the film, the rougher is its surface as depicted from the results on Table 4. Thus, in order to reduce the film rugosity, one should reduce the total amount of solid deposited. High rugosity might also mean a larger surface area to interact with the microbes and higher activity against the microbes.

As previously described, high conversion rates for the NPs synthesis depended on the presence of stabilizers, such as the amphiphiles used in this work, with the function of stabilizing the droplet/water interface during the polymerization [17]; only about 10% of the monomer mass added was converted into polymer in absence of the emulsifiers [19]. Here 2 mM amphiphile effectively lead to ≥80% monomer-to-polymer conversion but this was at the expenses of the core-shell nanoparticle structure; the PDDA shell around each PMMA core was practically lost due to the presence of amphiphile at the core-water interface of the NPs.

**Figure 8:**
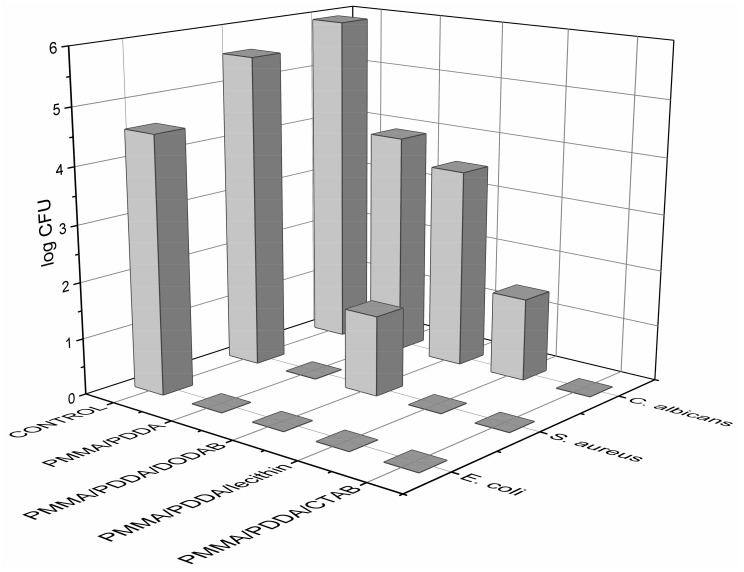
Microbicidal activity of PMMA/PDDA coatings on glass coverslips obtained from casting and drying under vacuum 50 µL of NPs dispersions in water obtained from 0.56 M MMA, 5 mg·mL^−1^ PDDA, 2 mM amphiphile CTAB or DODAB or lecithin during the polymerization and exhaustively dialysed thereafter. Since 5 mg·mL^−1^ of PDDA was used for NPs synthesis, in 0.05 mL of each dispersion used for the coatings, the PDDA amount active against microbes would be 0.25 mg. However, after dialysis, the final volume of the dialyzed dispersion increased inside the dialysis bags and diluted the dispersions by 10 to 12 (PMMA/PDDA), 10 to 19 (PMMA/PDDA/CTAB), 10 to 17 (PMMA/PDDA/DODAB), and 10 to 18 (PMMA/PDDA/lecithin); final PDDA amount in each coating was 0.210, 0.130, 0.145, and 0.140 mg, respectively.

Coatings from the NPs were also tested for their antimicrobial activity. The coatings contained about 0.20 mg of PDDA and reduced cell viability to zero in many instances (Figure 8). The PMMA/PDDA/CTAB coatings yielded a larger reduction in final CFU countings for the three microorganisms tested at 0.18 mg of PDDA (Figure 8). The low NPs size and high rugosity of the coatings (R) also affected the activity. The roughest coating cast from the smallest NPs was the one with the highest activity, namely the coating cast from the PMMA/PDDA/CTAB NPs (Table 4; Figure 8).

At 2 mM lecithin, since lecithin contains phospholipids and fatty acids [32,38], a net negative charge (zeta-potential equal to −21 mV) was obtained for PMMA/lecithin NPs synthesized in absence of PDDA even after exhaustive dialysis [17]. At 2 mM DODAB or CTAB, the PMMA/DODAB or PMMA/CTAB NPs exhibited positive zeta-potentials [17]. However, the lower zeta-potential for PMMA/CTAB in comparison to the one for PMMA/DODAB NPs was deemed consistent with the reported affinity of DODAB for the PMMA polymeric matrix that did not occur for CTAB—CTAB diffused to the outer water medium from PMMA coatings [27,28]. In summary, although the three stabilizers indeed improved monomer-to-polymer conversion, PDDA imparted the electro-steric repulsion between the MMA droplets during NP synthesis and represented an additional stabilizing factor for the PMMA/PDDA NPs. The use of cationic stabilizers such as DODAB and CTAB reduced PDDA shell surrounding the NPs (Figure 2).

**Figure 9:**
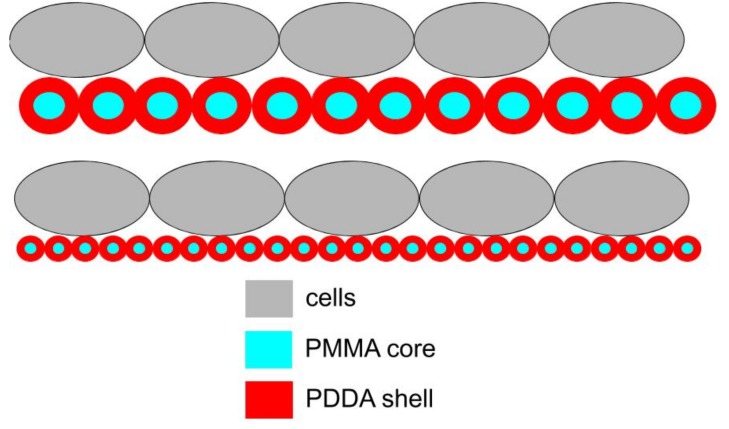
Scheme for the interaction between cells and coatings from PMMA/PDDA NPs illustrating a possible role of nanometric particle size on optimization of antimicrobial activity due to increase in the multipoint interaction between PDDA shell (in red) and cells (in grey). High rugosity for the films might also contribute to the increased surface area on the coating favouring the interaction with the microbial cells.

In this work, one of the most interesting findings referred to the role of NPs size on the microbicidal activity. Figure 9 illustrated this finding showing that the frequency of multipoint attachment of the cells to the coatings cast from NPs can increase with the decrease in NPs size. Thereby the coatings made from small PMMA/PDDA/CTAB NPs would be more effective in killing the cells than those made from large PMMA/PDDA/DODAB or PMMA/PDDA/lecithin NPs. 

The reason for the small size of PMMA/PDDA/CTAB NPs in the dispersion can be related to the conical shape of the CTAB molecule, which favors high curvature for the MMA droplets during polymerization. The lipids DODAB or lecithin with molecular shapes closer to cylinders would not favor the curvature of the MMA droplets so that final particle size would be larger than the one for stabilizers with a conical shape.

By adding amphiphiles such as CTAB, DODAB and lecithin as surfactants, active as stabilizers during the NPs synthesis, a major question was raised regarding the effect of the stabilizers on the core-shell NPs structure. Would the quaternary ammonium, cationic amphiphiles hamper the location of cationic PDDA as a shell surrounding each PMMA NP core? Apparently, this indeed happened since the shell thickness was substantially reduced in the dispersions with the cationic amphiphiles as shown in Figure 2 and Table 1. Using the cationic amphiphiles as stabilizers, changed PDDA location from the NP shell to bulk water phase. This reduced the PDDA shell surrounding NPs PMMA core. The negatively charged lecithin possibly found also as closed bilayers in dispersion also might have withdrawn PDDA from the NPs shell to a certain extent thereby reducing the shell thickness (Figure 2; Table 1).

Other questions referred to the eventual contribution of CTAB or DODAB to the antimicrobial activity of the dispersions and coatings. The reason for this question was the reported activity of DODAB and CTAB as antimicrobial agents [8,14,16,29,39,40,41,42]. In this work, the antimicrobial properties of these ternary systems both as latexes dispersions in water and as coatings were determined. PDDA resulted in the most important microbicidal agent due to its location as an outer shell of the core-shell PMMA/PDDA NPs and/or its release to the water medium of amphiphile stabilized NPs. Residual CTAB or DODAB would either remain in the NPs polymer matrix or leach from the NPs and through the dialysis membrane leaving the dispersion during dialysis and barely contributing to the microbicidal activity (Figure 5). PDDA did not leave the dispersions by dialysis so that free PDDA antimicrobial activity remained significant for dialyzed dispersions and their coatings.

## 3. Materials and Methods

### 3.1. Materials

MMA, PDDA, azobisisobutyronitrile (AIBN), NaCl, CTAB, DODAB, soybean lecithin, chloroform, agarose, and Mueller Hinton agar (MHA) were from Sigma-Aldrich (Darmstadt, Germany) and used without further purification. PDDA molecular weight was 100,000–200,000 g and came as a water solution at 20% PDDA. The composition of soybean lecithin includes several fatty acids and phospholipids [33,34]. Silicon <100> wafers were from Silicon Quest (USA) with a native oxide layer approximately 2 nm thick and used as substrates for casting the dispersions. The Si wafers with a native SiO_2_ layer were cut into small pieces of ca 1 cm^2^, cleaned with ethanol, and dried under an N_2_ stream; they are smooth substrates for the coatings. The syntheses in 1 mM NaCl solution prepared with Milli-Q water yielded NPs dispersions by emulsion polymerization that underwent dialysis for purification using a cellulose acetate dialysis bag with molecular weight cut-off around 12,400 g/mol. All other reagents were analytical grade and used without further purification.

### 3.2. Preparation of Hybrid Nanoparticles (NPs) by Emulsion Polymerization

A variety of hybrid and polymeric NPs were obtained by polymerization of MMA at 70–80 °C for 1 h using 10 mL of aqueous solutions of NaCl 1 mM and PDDA and/or CTAB, DODAB or lecithin in accordance with compositions shown in Table 5 [17,19]. Briefly, a weak flux of nitrogen was applied to the solution during a few minutes before adding 0.0036 g of AIBN initiator and MMA. For dispersions containing surfactants or lipids, DODAB or lecithin was previously dissolved in chloroform in order to prepare lipid films under a nitrogen flux to evaporate the chloroform solvent [43,44]. 10 mL of the NaCl 1 mM solution was then added to the dried lipid films before proceeding with the NPs synthesis. In the case of CTAB, the required amount of CTAB in the NPs dispersion was directly added to the 1 mM NaCl solution before starting the NPs synthesis. NPs dispersions thus obtained were further purified by dialysis against Milli-Q water until water conductivity reached 5 µS/cm.

### 3.3. Determination of Zeta-Average Diameter (Dz), Polydispersity (P), and Zeta-Potential (ζ) for PMMA/PDDA NPs Dispersions

Size distributions, Dz, ζ, and P were checked for NPs dispersions by dynamic light-scattering (DLS) using a Zeta Plus–Zeta Potential Analyzer (Brookhaven Instruments Corporation, Holtsville, NY, USA) equipped with a laser of 677 nm with measurements at 90° (Table 1). P of the dispersions was determined by DLS following well defined mathematic equations [45]. Dz values were obtained from the log normal distribution of the light -scattered intensity curve against the diameter. ζ values were determined from the electrophoretic mobility (μ) and Smolukowski equation ζ = μη/ε, where η and ε are the viscosity and the dielectric constant of the medium, respectively. Samples were diluted 1:30 with a 1 mM NaCl water solution for performing the measurements at (25 ± 1) °C.

### 3.4. Preparation and Physical Characterization of Coatings from the NPs Dispersions by Casting from Photographs and Contact Angle Determinations

Films prepared by casting employed 0.05 mL of PMMA/PDDA, PMMA/CTAB/PDDA, PMMA/DODAB/PDDA, PMMA/lecithin/PDDA dispersions (Table 1 and Table 3) casted onto three different surfaces: polystyrene, silicon wafers, or glass coverslips. After drying overnight under vacuum the films were photographed and characterized regarding their wettability. For determining antimicrobial activity films were cast onto glass coverslips. Wettability was evaluated by using a home build apparatus as previously described [46,47]. Sessile water droplets of 10 µL allowed determining the advancing contact angle (Ө_A_) over the first five minutes after depositing the droplet on the films.

### 3.5. Microorganisms Growth and Determination of Cell Viability in the Presence of the PMMA/PDDA Dispersions and Coatings

*E. coli* ATCC (American Type Culture Collection) 25322, *S. aureus* ATCC 29213 and *Candida albicans* ATCC 90028 growth was from previously frozen stocks kept at −20 °C in the appropriate storage medium. The bacterial strains plated onto MHA were incubated at 37 °C/18–24 h before transferring some isolated colonies to an isotonic 0.264 M D-glucose solution and adjusting turbidity to 0.5 of the McFarland scale [48]. The 0.264 M D-glucose solution was used instead of any culture medium because cationic molecules are inactivated by the relatively high ionic strength or negatively charged molecules such as amino acids and polysaccharides. For determination of cell viability after interaction with the NPs, 0.1 mL of the cell suspensions (around 10^8^ CFU/mL) were mixed with 0.9 mL of NPs dispersions diluted in the same D-glucose solution. Cell viabilities (log CFU/mL) were plotted as a function of PDDA concentration (mg·mL^−1^).

60 µL of the bacterial suspensions were deposited on the coatings (obtained by casting of dispersions described on Table 3 and Table 4 onto glass coverslips) and left in a water vapour saturated chamber for 1 h to prevent water evaporation from the droplet. Thereafter, the glass coverslips were transferred to 10 mL of 0.264 M D-glucose isotonic solution in Falcon tubes and vigorously stirred by vortexing before withdrawing 0.1 mL aliquots and preparing their 1:10 and 1:100 dilutions for plating on MHA plates, incubating the plates (37 °C/24 h) and counting the CFU. These readings were converted into CFU/mL and log (CFU/mL). When no counting was obtained, since the log function does not exist for zero, the CFU/mL counting was taken as 1 so that log CFU/mL could be taken as zero. Controls were bare glass coverslips.

### 3.6. Determination of Inhibition zones from NPs Dispersions and/or their Supernatants

*S. aureus* ATCC 29213 from previously frozen stocks kept at −20 °C in an appropriate storage medium was grown as described above and the bacterial suspension prepared in 0.264 M D-glucose had its turbidity adjusted according to 0.5 of the McFarland scale as previously described. 

A growth medium containing 2.3% Muller-Hinton broth and 0.64% agar was prepared and sterilized. Into 50 mL of this growth medium, 0.5 mL of the *S. aureus* suspension was added and then carefully homogenized. Plates containing MHA were previously prepared and used to place micropipette tips with their bases positioned on the MHA in order to form wells with 9mm diameter where each antimicrobial sample can be applied, after pouring ca. 20 mL of the *S. aureus* culture. After agar hardening, the tips were removed and, in each well, 70 µL of the PDDA solutions at 0.01, 0.1, 0.2, 0.5, 1, 1.5, 2, and 2.5 mg·mL^−1^ PDDA or of the NPs dispersions were added.

### 3.7. Determination of PDDA by Chloride Microtitration

Chloride, as the PDDA counterion, was microtitrated to determine [PDDA] in mg·mL^−1^ after obtaining a standard curve for the linear dependence between PDDA and chloride concentrations. The method was previously described by Schales and Schales [49]. The detailed protocol for the halide microtitration can be found in reference [50].

## 4. Conclusions

The ternary systems composed of PMMA/PDDA/amphiphile NPs evaluated in dispersion or as coatings obtained by casting onto three different substrates displayed variable but important antimicrobial activity against three different pathogenic microbes, *E. coli*, *S. aureus* and *C. albicans.*

The macroscopic aspects for the coatings revealed homogeneous films on the hydrophilic silicon wafers or glass coverslips and cracked non-adherent coatings on the hydrophobic polystyrene surfaces. The amphiphiles employed during NPs synthesis changed the wettability of the coatings yielding contact angles that increased with the increase in the hydrophobicity of the amphiphile employed; thereby the lowest contact angle occurred in absence of amphiphile whereas the highest one occurred with DODAB as the most hydrophobic amphiphile as compared to CTAB or lecithin. At about 0.140 mg of PDDA in PMMA/PDDA/amphiphile dispersions casted and dried on the substrates, the coating with higher microbicidal activity was the one obtained from PMMA/PDDA/CTAB dispersions because the small NP size favoured both high frequency of contacts between PDDA in the outer NP medium for contacting the microbial cells; all pathogens present (10^4^–10^6^ CFU) completely lost their viability upon interaction with these coatings for 1 h.

The microbicidal activity of the ternary dispersions determined from cell viability curves over a broad range of PDDA concentrations revealed that the most sensitive microorganism to the dispersions was *E. coli,* followed by *S. aureus* and *C. albicans.* The PMMA/PDDA/CTAB dispersions were the most effective in killing the three microbial strains tested by 7–8 logs in 1 h. The nanometric and cationic NPs (sizes below 100 nm) of PMMA/PDDA/CTAB yielded maximal microbicidal activity either as dispersions or coatings. There were 6–8 logs reduction of cells viability from [PDDA] ≥ the minimal microbicidal concentration.

All NPs exhibited hydrodynamic diameters of about 200 nm with exception of the PMMA/PDDA/CTAB NPs with 90 ± 1 nm as Dz; this size allowed NPs penetration through the cell walls and cell membrane with significant damage to both structures and complete loss of cell viability at 0.030, 1.000, and 1.310 mg·mL^−1^ PDDA carried by the PMMA/PDDA/CTAB NPs against *E. coli, S. aureus* and *C. albicans*, respectively. In summary, in this work the presence of residual amphiphile stabilizers in PMMA/PDDA/amphiphile NPs revealed their important role during the emulsion polymerization that allowed high monomer-to-polymer conversion; however, the significant microbicidal agent in these NPs was only one, the cationic antimicrobial polymer PDDA, which exhibited the potency required to kill microbes over 6–8 logs of CFU countings.

The reduction in particle size and increased rugosity and surface area for the coatings were associated with CTAB and its molecular geometry. CTAB promoted the synthesis of the smallest NPs. Their casting onto hydrophilic surfaces generated coatings with the highest rugosity and microbicidal activity.

Finally, all three amphiphiles negatively affected the core-shell NPs structure, reducing the thickness of the shell in the core-shell NPs and releasing PDDA to the medium.

## Figures and Tables

**Table 1:** NPs physical properties as determined for dispersions aged 180 days. The mean hydrodynamic diameter (Dz), the polydispersity (P) and the zeta-potential (ζ) were obtained by dynamic light scattering (DLS). The mean diameter (D) was obtained for dry dispersions from scanning electron microscopy (SEM) and sizing analysis by the ImageJ software. The Dz_minimum_ was obtained from the effect of [NaCl] on Dz in Figure 2. The NP shell thickness is ∆/2.

Dispersion	D/nm	Dz/nm	Dz_minimum_/nm	∆/2/nm	P	ζ/mV	Solids/mg·mL^−1^	Conversion/%
PMMA/PDDA	164 ± 32	226 ± 3	166 ± 2	30	0.010 ± 0.010	51 ± 1	6 ± 1	11 ± 1
PMMA/PDDA/CTAB	97 ± 14	116 ± 1	98 ± 1	9	0.040 ± 0.010	50 ± 2	26 ± 1	79 ± 1
PMMA/PDDA/DODAB	183 ± 28	226 ± 1	194 ± 1	16	0.030 ± 0.020	54 ± 1	17 ± 1	47 ± 1
PMMA/PDDA/lecithin	190 ± 25	217 ± 2	194 ± 1	11.5	0.040 ± 0.020	55 ± 1	8 ± 1	24 ± 1

**Table 2:** Microbicidal activities of PDDA, PMMA/PDDA and PMMA/PDDA/amphiphile dispersions plotted as minimal microbicidal concentrations (MMC).

Dispersion ^1^	Minimal Microbicidal Concentration (MMC), mg·mL^−1^		
	*E. coli*	*S. aureus*	*C. albicans*
PDDA ^2^	0.005	>0.500	>0.005
PMMA/PDDA ^2^	0.007	>0.500	>1.000
PMMA/PDDA/DODAB	0.100	1.000	>3.000
PMMA/PDDA/CTAB	0.030	1.000	1.310
PMMA/PDDA/Lecithin	0.250	0.550	>3.000

^1^ Concentrations used for NPs synthesis were: [MMA] = 0.56 M; [PDDA] = 5 mg·mL^−1^; [CTAB] = [DODAB] = [Lecithin] = 2 mM; ^2^ Data from reference [19].

**Table 3:** PDDA concentration in the supernatant of NPs dispersion after dialysis. The experimental error for determining [PDDA] was around 10% of the mean values shown on the table.

Dispersion	[PDDA]_total_/mg·mL^−1^	[PDDA]_supernatant_/mg·mL^−1^ Micro-Titration	[PDDA]_supernatant_/mg·mL^−1^ Inhibition Zone	[PDDA]_nanoparticles_/mg·mL^−1^
PMMA/PDDA	4.2	3.2	3.5	0.7–1.0
PMMA/PDDA/DODAB	2.9	2.5	2.8	0.1–0.4
PMMA/PDDA/CTAB	2.6	1.9	2.8	0.0–0.7
PMMA/PDDA/lecithin	2.8	2.9	3.5	0.0–0.0

**Table 4:** Physical properties of PMMA/PDDA/amphiphile coatings on silicon wafers. Casting of PMMA/PDDA/CTAB, PMMA/PDDA/DODAB, or PMMA/PDDA/lecithin NPs dispersions followed by drying under vacuum was compared to spin-coated [28] and PMMA/PDDA films [17].

Materials	Procedure	Thickness/µm	Rugosity R	Refractive Index	Contact Angle Ө_A_/°
PMMA ^1^	Spin-coating	0.091 ± 0.001	N.A.	1.4999 ± 0.004	76 ± 5
PMMA/PDDA ^1^	Spin-coating	0.094 ± 0.003	N.A.	1.4951 ± 0.004	15 ± 1
PMMA/PDDA ^2^	Casting	3.82	1.6	N.A.	9 ± 2
PMMA/PDDA/CTAB	Casting	12.70	4.0	N.A.	14 ± 1
PMMA/PDDA/DODAB	Casting	4.32	2.2	N.A.	47 ± 3
PMMA/PDDA/lecithin	Casting	9.17	3.0	N.A.	25 ± 1

^1^ Data from reference [28]; ^2^ data from reference [17]; N.A. means non-available.

**Table 5:** Concentrations of MMA, PDDA, CTAB, DODAB and/or lecithin used to synthesize hybrid NPs by emulsion polymerization. The final PDDA concentrations departed from the expected value of 5 mg·mL^−1^ due to water entering the dialysis bag during dialysis. This was corrected to dilution after measuring the final volume of the dialyzed dispersion since the dispersions were used always after dialysis.

Dispersion	[MMA]/M	[PDDA]/mg·mL^−1^	[PDDA]_corrected_/mg·mL^−1^	[CTAB]/mM	[DODAB]/mM	Lecithin/mM
PMMA/PDDA	0.56	5	4.2	-	-	-
PMMA/PDDA/CTAB	0.56	5	2.6	2	-	-
PMMA/PDDA/DODAB	0.56	5	2.9	-	2	-
PMMA/PDDA/lecithin	0.56	5	2.8	-	-	2

## Abbreviations

NPs	Nanoparticles
PMMA	poly (methylmethacrylate)
PDDA	poly (diallyldimethylammonium) chloride
CTAB	cetyltrimethylammonium bromide
DODAB	dioctadecyldimethylammonium bromide
CFU	colony forming unities
MMA	Methylmethacrylate
Ө_A_	advancing contact angle
AIBN	Azobisisobutyronitrile
Dz	zeta-average diameter
P	Polydispersity
ζ	zeta-potential
MHA	Mueller-Hinton agar
DLS	dynamic light scattering
μ	electrophoretic mobility
η	viscosity of the medium
ε	dielectric constant of the medium
ATCC	american type culture collection
